# Not all Sicca is Sjögren's and not all Sjögren's is Sicca

**DOI:** 10.7759/cureus.12996

**Published:** 2021-01-29

**Authors:** Melissa Neumann, Javier Quintero, Tiffany Shih, Eugenio M Capitle

**Affiliations:** 1 Internal Medicine, Rutgers University, Newark, USA; 2 Rheumatology, Rutgers University, Newark, USA

**Keywords:** sjogren and facial palsy, facial nerve palsy and sjogren, sjogren and sicca

## Abstract

Symptoms of dry eyes or dry mouth, otherwise known as sicca symptoms, are not always present in patients with Sjögren's syndrome (SS). Approximately 20% of patients with SS do not have sicca symptoms. An unusual case of a patient presenting with complete left-sided facial hemiparesis, a history of partial bilateral sensorineural hearing loss who was found to have elevated antinuclear antibody (ANA) with high titer positive SSA/Ro antibody, evidence of bilateral parotitis on imaging and absence of sicca symptoms, prompted us to perform a literature review. Twelve case reports relating facial nerve palsy and Sjögren's were found and only one described a similar constellation of features of unilateral facial weakness and otalgia. Management of facial nerve palsy related to Sjögren's is unclear but pharmacological agents have included corticosteroids, intravenous immune globulin (IVIG), cyclophosphamide, and plasmapheresis. This case report describes a patient whose facial nerve palsy is attributed to SS, explores peripheral and central nervous system involvement in SS, and provides some recommended treatments.

## Introduction

Symptoms of dry eyes or dry mouth, otherwise known as sicca symptoms, are not always present in patients with Sjögren’s syndrome (SS). Approximately 20% of patients with SS do not have sicca symptoms. This case report highlights a patient with no sicca symptoms presenting with facial nerve palsy and meeting Sjögren’s diagnostic criteria. Twelve case reports relating facial nerve palsy and Sjögren’s were found in the literature. This case report describes a patient whose facial nerve palsy is attributed to SS, explores peripheral and central nervous system involvement in SS, and provides some recommended treatments. 

## Case presentation

A 62-year-old female presented to the rheumatology clinic with complaints of left-sided facial weakness. Facial weakness was of sudden onset and had persisted for the past two months. She was initially seen by her primary care physician (PCP) who empirically treated for Bell’s palsy with seven days of prednisone 20 mg and acyclovir without response. She was then tried on a two-week tapering regimen starting with prednisone 60 mg which, again did not help subside the symptoms. Of note, this was her second episode of left-sided facial weakness; she had a similar episode 15 years prior that self-resolved.

Her past medical history included partial bilateral sensorineural hearing loss and hypertension. Home medications included olmesartan, a baby aspirin, and her recent steroid regimen. She has a daughter with systemic lupus and lupus nephritis. The patient works as a nurse practitioner and is originally from the Philippines. She is a lifetime nonsmoker and denied any recent travel, sick contacts, prolonged outdoor activities, or bug bites.

The patient denied history of trauma, recurrent fevers, skin rash, weight loss, blurry vision, headaches, sore throat, dysphagia, nasal/oral sores, recurrent caries, dry eyes, or dry mouth. She denied any limb weakness, numbness, or paresthesias. Also, she denied joint pain, swelling, Raynaud's phenomenon, h/o miscarriages, dyspnea, alopecia, cough, chest pain, palpitations, or abdominal symptoms.

Physical exam was remarkable for bilateral facial fullness and complete left-sided facial hemiparesis including upper and lower face with inability to wrinkle her forehead. Extraocular movements were intact. No oral or nasal lesions were noticed. She had no palpable cervical or axillary adenopathy. Chest and cardiovascular exam were unremarkable. There was no palpable organomegaly on abdominal exam. Strength of upper and lower extremities was 5/5 bilaterally. No skin rash was noticed. All vital signs were within normal range.

Investigative workup was remarkable for negative studies for herpes simplex virus (HSV), Lyme disease, and HIV. Serological testing revealed a positive low titer antinuclear antibody (ANA) 1:80 in speckled pattern with a high titer SSA/Ro antibody. Her blood cell counts, complete chemistry profile, thyroid testing, HgbA1C, erythrocyte sedimentation rate, C-reactive protein, and serum protein electrophoresis were within normal range. Infectious workup was also unremarkable for syphilis, tuberculosis, or viral hepatitis.

**Figure 1:**
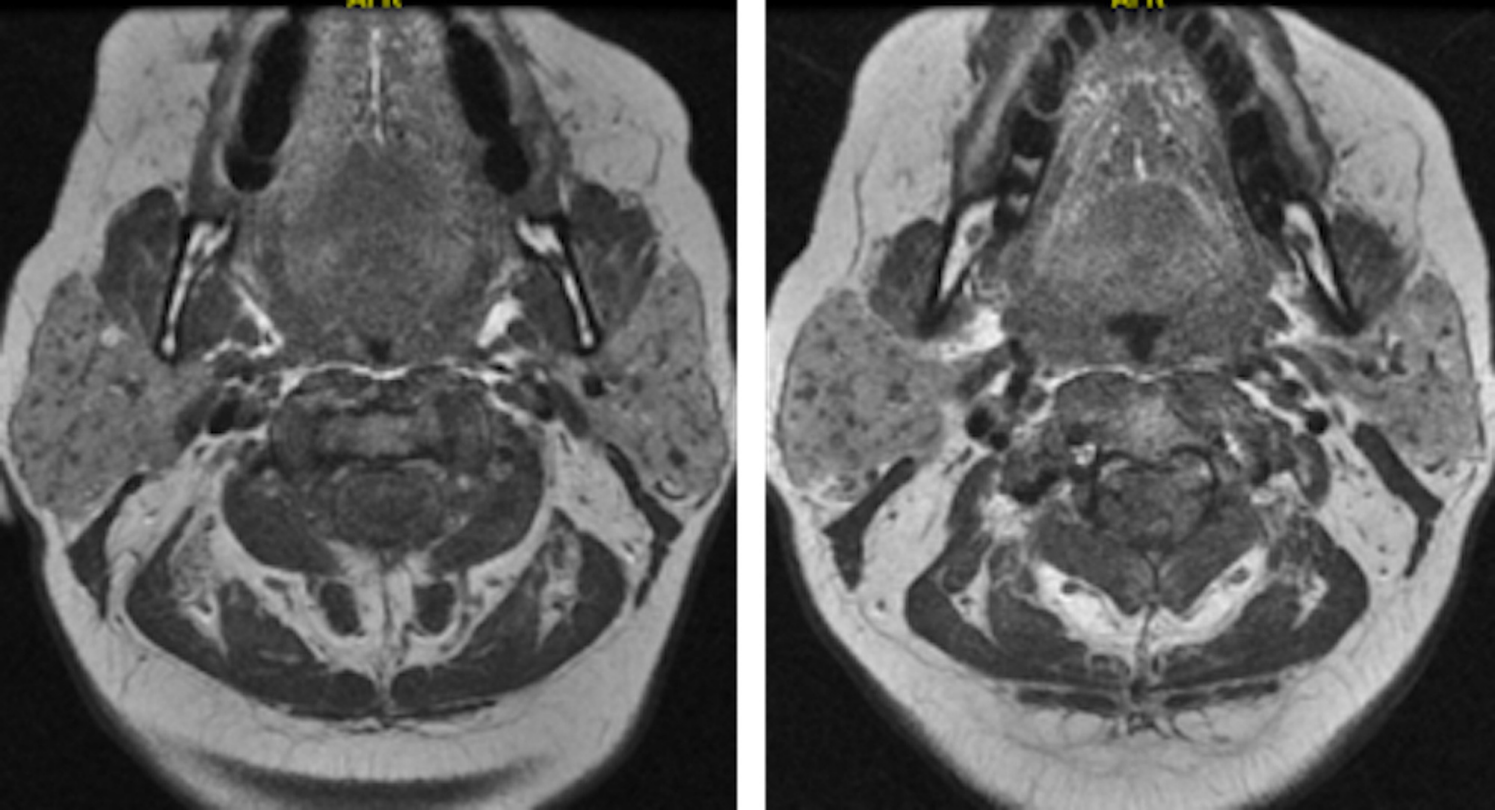
MRI brain with and without contrast. T1-Weighted images in sequential order craniocaudally (right to left image) shows enlarged parotid glands bilaterally, more pronounced on right side with nodular ductal dilations and mixed calcifications. This MRI also shows contrast enhancement of the tympanic segments of bilateral facial nerves, compatible with facial neuritis.

Due to the persistent facial nerve palsy, the patient underwent an MRI of her brain and internal auditory canal with and without contrast. The results showed contrast enhancement of the tympanic segments of bilateral facial nerves, compatible with facial neuritis. The MRI also showed enlarged parotid glands bilaterally, more pronounced on the right side with ductal dilatation and mixed calcifications consistent with bilateral parotitis. Images are shown in Figure 1. Chest X-ray showed increased interstitial markings but no hilar lymphadenopathy or effusion. Subsequent CT of the chest was negative for interstitial lung disease or lymphadenopathy. Based on high index of clinical suspicion, supported by imaging and serological workup, the patient was diagnosed with facial nerve palsy secondary to SS. After thorough workup and evaluation, she was started on immunosuppressive therapy with mycophenolate mofetil and prolonged steroid taper with significant clinical improvement.

 

## Discussion

Sjögren’s syndrome is a chronic, systemic, autoimmune disease of the exocrine glands, which often affects the lacrimal and salivary glands. The prevalence of SS is difficult to determine because of frequent changes in diagnostic criteria; however, it is estimated to be 1% (0.1%-4.8%) [1]. SS is the second most common autoimmune disease, after rheumatoid arthritis, and typically affects middle-aged females, with a mean age of onset in the fourth or fifth decade [1]. ANAs are the most frequent autoantibodies detected, with anti-Ro/SS-A antibody as the most specific antibody. Cryoglobulins and hypo-complementemia are the main prognostic markers.

Despite its classic association with dry eyes and dry mouth syndrome, approximately 20% of patients with SS do not have sicca symptoms. Most of these patients may not ever develop sicca symptoms as part of their disease manifestations but may have other features of systemic involvement across 12 different disease domains described in the EULAR Sjögren’s Syndrome Disease Activity Index (ESSDAI). Moreover, only 10% of patients with clinically significant dry eye syndrome have SS [1]. It was demonstrated by Baer et al. in 2015 that the presence of anti-La/SS-B antibodies alone lacked specificity for the diagnosis of SS [2]. The most recent classification criteria for SS (2016 ACR/EULAR) are based on the weighted sum of factors such as unstimulated whole saliva flow rate, abnormal Schirmer test, abnormal Ocular staining score, presence of focal lymphocytic sialadenitis score on salivary gland biopsy, and presence of anti-Ro/SSA antibodies. Although this classification criteria may not include abnormal imaging of salivary glands, experts propose that certain findings on imaging may also be included as diagnostic consideration. This is especially true when patients have evidence of parotitis on MRI, salivary ultrasound, CT, or salivary scintigraphy.

When a patient presents with neurological involvement as the initial presentation of SS, other primary neurological conditions should be excluded. Neurological disorders are one of the most common extra-glandular features of SS, affecting approximately 8.5%-70% of patients. As seen in our patient, neurological symptoms may precede SS diagnosis in 25%-60% of cases by two years on average, up to 12 years [3]. Neurological manifestations of SS may vary, affecting both the peripheral (PNS) and central nervous system (CNS) with an overall prevalence of 5%-20%. PNS disease is a well-documented feature of SS, presenting in about 25% of patients, most commonly as peripheral neuropathy. The presence of peripheral neuropathy in SS decreases the quality of life and is a negative prognostic factor. Although the pathogenesis is not fully understood, it is believed to involve T lymphocytes and dendritic cells secretion of inflammatory cytokines leading to vasculitis and damage to the dorsal root ganglia.

Central nervous system (CNS) involvement is much less common and may be seen in 1%-5% of SS patients. In the past decade, CNS involvement, such as the brain and spinal cord, has been observed more commonly than initially suspected. Various symptoms have been reported which are connected with cranial and autonomic nervous system damage as the initial primary presentation of SS. The underlying mechanism of CNS involvement in SS has been hypothesized to be due to three possible factors: direct infiltration of the CNS by mononuclear cells, vascular involvement related to antineuronal antibodies, or ischemia secondary to small vessel vasculitis [4]. 

Multiple cranial neuropathies, a manifestation of PNS involvement, is less frequently seen. Cranial nerve (CN) involvement has a female to male ratio of 20.8:1. Interestingly, as many as six cranial nerves can be involved simultaneously, like our patient with a history of sensorineural hearing loss (SNHL) implying CN VIII dysfunction, and now facial nerve palsy (i.e. CN VII involvement) [5]. Although the most common presentation of CN involvement is that of the trigeminal nerve (CN V), other cranial nerves have been described including the facial nerve (VII, causing facial nerve palsy); the vestibulocochlear nerve (VIII, causing neural deafness and vestibular dysfunction); and the oculomotor nerve, trochlear nerve, or abducens nerve (III, IV, and VI respectively, causing diplopia). Multifocal involvement of CN III, V, VI, VII, IX, X, and XII can occur in multiple combinations [6]. Some 25% of SS patients will experience SNHL at some point during their disease process.

This patient presented with recurrent and now persistent left facial nerve palsy. When evaluating a patient with facial nerve palsy, we can consider different causes such as a cerebrovascular accident, otitis media, diabetes mellitus, sarcoidosis, HSV, HIV, Lyme disease, Guillain-Barré syndrome, mass effect causing nerve compression (parotid neoplasms, acoustic neuroma), or trauma (temporal bone fractures, post-surgical). Ultimately, most patients are determined to have idiopathic facial nerve palsy, also known as Bell’s palsy. Onset of symptoms in Bell’s palsy typically occurs over one to two days, with maximum paralysis by three weeks, and recovery in less than six months. If symptoms have not resolved within four months, Bell’s palsy is unlikely, and a secondary etiology for facial paralysis should be explored.

The most common rheumatic disease associated with facial nerve palsy is SS. The facial nerve contains motor fibers (innervating the facial muscles), parasympathetic fibers (innervating lacrimal, submandibular, and sublingual salivary glands), afferent fibers (receptors from anterior two-thirds of the tongue), and somatic afferents (receptors from external auditory canal and pinna). The facial nerve also supplies the lacrimal and palatine glands via the greater petrosal nerve, which branches off the facial nerve at the geniculate ganglion. Facial nerve palsy presents in a lower motor neuron pattern, manifesting as unilateral facial weakness, as was seen in our patient. Unlike lesions affecting the PNS (lower motor neurons), lesions involving the CNS (central brain/brainstem) would still maintain upper facial (forehead) motor function. Diagnostic testing may include electrodiagnostic studies to localize the lesion, such as electromyography (EMG) or nerve conduction studies (NCS). Imaging studies (like CT or MRI) may be warranted in patients with persistent symptoms. Neurophysiological studies can be used for prognostication of facial nerve palsy. A reduction of nerve conduction signal by less than 90% compared to the contralateral side, portends a favorable prognosis.

Mori et al. performed a study of 92 patients with SS-associated neuropathy [6]. A striking finding is that in 93% of the cases, symptoms of neuropathy actually preceded the diagnosis of SS, similar to our patient. Interestingly, for cranial neuropathies, only 0-40% of patients were positive for anti-SS-A and anti-SS-B antibodies. The authors attribute the sensory neuropathy to degeneration of neurons and mononuclear cell infiltration without vasculitis, suggesting ganglion neurons being targets of SS. On the other hand, the cranial neuropathies are more closely associated with a vasculitic process with subsequent axonopathy.

Facial nerve palsy due to SS is unusual previously described in 12 case reports before this one. Among the initially reported cases, Henrik Sjögren described a patient in 1935 with bilateral facial palsy and was the first to mention cranial neve involvement in SS patients [7]. All of the published case reports of facial nerve palsy are female patients, ranging in age from 32 to 59 years old. Three cases (Sjögren et al., Uchihara et al., and Wei et al.) describe bilateral facial palsy [7-9]. Colaci et al. and Mori et al. were more extensive scale studies [3, 6]. Mori et al. reviewed 92 patients with SS and associated neuropathy; 10 of these patients had facial involvement [6]. 

 One patient had bilateral facial palsy without any other cranial nerve involvement. Colaci et al. performed a mega study finding 10 cases with involvement, including facial palsy [3]. No specific details were provided for these patients with facial palsy. Two of the remaining cases had other conditions that the authors believe may have contributed to facial palsy. Hadithi et al. describes a patient with facial palsy and concurrent autoimmune hypothyroidism [10], and Rousso et al.’s patient had a concurrent B12 deficiency [11]. Birnbaum et al. found two cases of Sjögren's and facial palsy, both characterized by facial weakness, otalgia, and hemifacial spasm [12]. The author proposes that this constellation of symptoms is unique to rheumatic disease and is separate from Bell palsy, which does not cause hemifacial spasm, otalgia, and severe neuropathic pain. A ninth case (Ashraf et al.) ascribes it to vasculitic damage of the vasa nervosum as the cause of facial palsy and other CNs palsies (VIth, VIIIth, IXth, Xth, and XIIth) [13]. The 10th case (Berault-Dupont et al.) of right facial paralysis ascribes the cause to SS [14].

Our case report is most similar to Birnbaum et al., which describes a similar constellation of features of unilateral facial weakness and otalgia [12]. Our patient’s only pre-existing condition was hypertension, treated with olmesartan. She had no other medical complications, such as thyroid disease, diabetes, HSV, HIV, or B12 deficiency. We also ascribe the etiology of our patient’s facial palsy to SS. Our patient also had a history of bilateral SNHL years prior to facial palsy. We attribute our patient’s facial palsy and SNHL to PNS involvement of SS, which was supported by MRI showing enhancement of facial and tympanic nerves consistent with neuritis. Our patient’s bilateral sensorineural hearing loss is likely also due to PNS involvement, as described by Calzada et al. who attributes this to IgG deposition on the basement membrane of the stria vascularis [15].

Treatment

In general, the nervous system’s involvement in SS is considered a negative prognostic factor and is usually related to a more aggressive course of the disease. While the therapeutic management of SS has not changed substantially in recent decades, specific therapeutic targets beyond the relief of symptoms remain scarce. For example, treatment of small-fiber neuropathy is generally aimed at symptomatic relief and includes the use of medications such as gabapentin, pregabalin, and serotonin and norepinephrine reuptake inhibitor (SNRIs). However, a trial of immunomodulatory or immunosuppressive therapy is appropriate for those who fail symptomatic therapy, such as intravenous immunoglobulin (IVIG) in a dose of 0.4g/kg for five days. Involvement of multiple mononeuropathies, as in our patient, then immunosuppressive medications such as high-dose corticosteroids (equivalent to prednisone 1 mg/kg/day), and cyclophosphamide (oral or IV) are considered, with reported response rates as high as 100%.

A recently updated international collaborative study (EULAR SS Task Force) developed the first EULAR evidence and consensus-based recommendations for the management of patients with SS. The therapeutic approach follows the 2014 EULAR standardized operating procedures and includes three overarching, general consensus-based recommendations. These 12 specific recommendations form a logical sequence, describing the management of the central triplet of symptoms (dryness, fatigue, and pain) followed by the management of systemic disease. SS can be effectively treated with corticosteroid and immunosuppressive agents such as cyclophosphamide, which has been found to be efficacious in patients with myelopathy and multiple neuropathy. Cyclophosphamide has also been found to be efficacious in cases with vasculitis associated with SS. Other immunosuppressives such as azathioprine, methotrexate, and cyclosporine have shown variable efficacy [16]. The 2019 EULAR recommendations, suggest a multidisciplinary approach involving various health professionals with shared decision making based on the clinical presentation after careful organ-by-organ evaluation of both severity and organ damage [17]. In general, the management of systemic features in SS is advised to follow a schedule consisting of a two-stage sequential regimen with treatment for other systemic autoimmune diseases. This includes a first intensive immunosuppressive approach targeted to restore organ function and induce remission as soon as possible, followed by a second therapeutic course aimed at maintaining the initial therapeutic response. Damage to the nervous system should indicate more intensive treatment. Some patients have shown rapid and almost complete recovery from nerve palsy after therapy with corticosteroids and IVIG [3, 12]. This may suggest that lymphocytic infiltrate, rather than a vasculitic process, is likely the cause of cranial neuropathy in SS.

Within the case reports found, treatment for SS-neuropathy includes: a combination of corticosteroids and immunosuppressants, IVIG, plasmapheresis, D-penicillamine, and even infliximab [6]. In our literature review, Mori et al. used corticosteroids and IVIG for 92 patients, showing favorable response [6]. This study proposed that IVIG should likely be used for painful dysesthesias of painful sensory neuropathy and radiculoneuropathy. Successful treatment for facial palsy and SS, as described in the case reports, was focused mostly on IVIG and corticosteroids. IVIG was used successfully in Birnbaum et al.; where one patient was treated successfully with IVIG, and the other was enrolled in a clinical trial with baminercept resulting in the resolution of symptoms [12]. An inadequate response to methotrexate was reported in Wei et al. [9]. Corticosteroids successfully treated three patients (Uchihara et al., Rousso et al., and Ashraf et al.) [8, 11, 13]. Ashraf et al. described a 47-year-old female with multiple cranial nerve palsies, including right facial weakness, sensorineural deafness, and pachymeningitis [13]. Initial symptoms were successfully treated with IV methylprednisolone, followed by prednisone, and then methotrexate. This patient had a recurrence of transient nerve involvement, including dysphagia, diplopia, with right-sided IXth, Xth, and XIIth cranial nerve palsies. In Rousso et al., the patient was treated with vitamin B12 replacement as well as corticosteroids and hydroxychloroquine resulting in resolution of facial palsy [11]. Hadithi et al.’s patient had resolution of symptoms by treating her underlying thyroid disease with thyroxine supplement and artificial tears [10]. Berault-Dupont et al.’s patient had no resolution of symptoms with corticosteroids [14].

With the inconsistent efficacy of both corticoid and immunosuppressive drugs in SS, new studies have been undertaken. Disease-modifying therapies for SS has been more challenging than expected when compared with the relative success in rheumatoid arthritis and other systemic inflammatory diseases. Treatment of CNS and PNS involvement in SS still remains anecdotal and empirical, based on reports drawn by treating cerebral systemic lupus erythematosus (SLE). One study has shown that IVIG could benefit patients with CNS vasculitis associated with SS [18]. Interestingly, immunosuppressive treatment also seems to help reverse any dementia related to CNS-SS. Psychotropic medications such as tricyclic antidepressants are advised to be used with caution in patients as their anticholinergic properties may exacerbate mucosal dryness [19].

Large randomized controlled studies are still lacking, and most protocols used are mainly empirical. Currently, on clinicaltrials.gov there is an active clinical trial in France (last updated July 27, 2020) specifically for SS associated painful sensory neuropathies with experimental IVIG treatment in Phase III (NCT03700138). Medico-economic and quality of life impact of Sjögren-associated small fiber neuropathy is also currently under investigation in France (last updated January 10, 2020, NCT03509064).

Current challenges in clinical trials of new therapies for SS include difficulties in selecting a well-defined study population, limited sensitivity to change of patient-reported symptoms scales in assessment of treatment efficacy, and the identification of combination therapeutic targets for disease-modifying agents, including biologics. Hydroxychloroquine, rituximab therapy, lymphotoxin alpha/beta heterodimers, and abatacept have not been shown to be effective for SS. However, a small study of 11 patients with neurological involvement treated with rituximab, showed benefit for the one patient with cranial nerve VII involvement who had previously failed treatment with cyclophosphamide and azathioprine [20]. New studies are looking into biologics that target T-cells and intracellular signaling pathways, including B cell survivor factor (BAFF) pathway [17].

Recent 2019 EULAR recommendations mention short courses of glucocorticoids (prednisone 0.5-1 mg/kg/day) as first line treatment, and cyclophosphamide pulses remains a second line consideration. For refractory or life-threatening disease, rituximab with or without plasma exchange is an option, especially when neuropathy is associated with cryoglobulinemic vasculitis [17].

## Conclusions

Although sicca symptoms are associated with SS, sicca symptoms are not always present in patients with SS. This patient presented with recurrent and then persistent left facial nerve palsy and was found to have ANA titer of 1:320, positive rheumatoid factor (RF), positive anti-Ro/SSA antibodies and bilateral parotid gland involvement on MRI, ultimately attributing her facial nerve palsy to SS.

Currently classification criteria for SS does not include abnormal imaging results; however, experts stipulate SS can be suspected if certain findings on MR, Ultrasound, CT, salivary scintigraphy, or sialography are present. Diagnostic criteria for SS need to be officially established in order to facilitate a formal diagnostic process for physicians. This case report teaches us that persistent CN palsies should prompt consideration of autoimmune conditions such as SS despite absence of sicca symptoms. It also demonstrates that mycophenolate along with corticosteroids may be considered as good therapeutic options.
